# Prediction of absolute risk of fragility fracture at 10 years in a Spanish population: validation of the WHO FRAX ™ tool in Spain

**DOI:** 10.1186/1471-2474-12-30

**Published:** 2011-01-28

**Authors:** Rafael Azagra, Genís Roca, Gloria Encabo, Daniel Prieto, Amada Aguyé, Marta Zwart, Sílvia Güell, Núria Puchol, Emili Gene, Enrique Casado, Pilar Sancho, Sílvia Solà, Pere Torán, Milagros Iglesias, Victòria Sabaté, Francesc López-Expósito, Sergio Ortiz, Yolanda Fernandez, Adolf Diez-Perez

**Affiliations:** 1 Department of Medicine, Universitat Autònoma de Barcelona. Psg Vall d'Hebrón 119-129, 08035 Barcelona, Spain; 2 Primary Healthcare Centre Badia del Vallès, Catalan Health Institute. C/ Bética s/n, 08214 Badia del Vallès (Barcelona), Spain; 3 Doctorate Program, Department of Medicine, Universitat Autònoma de Barcelona. Psg Vall d'Hebrón 119-129, 08035 Barcelona, Spain; 4 Primary Healthcare Centre Sant Llàtzer, Sanitary Consortium of Terrassa. C/ de la Riba 62, 08221 Terrassa (Barcelona), Spain; 5 Nuclear Medicine Service, Vall d'Hebrón University Hospital. Psg Vall d'Hebrón 119-129, 08035 Barcelona, Spain; 6 Primary Healthcare Centre Passeig Sant Joan, Catalan Health Institute. Psg de Sant Joan 267, 08035 Barcelona, Spain; 7 Primary Healthcare Centre Granollers Centre, Catalan Health Institute. C/ Museu 19, 08400 Granollers (Barcelona), Spain; 8 Primary Healthcare Centre Can Gibert del Plà-Girona-2, Catalan Health Institute. C/ Sant Sebastià 50, 17005 Girona, Spain; 9 Primary Healthcare Centre Montcada i Reixach, Catalan Health Institute. Psg de Jaume I s/n, 08110 Montcada i Reixac (Barcelona), Spain; 10 Emergency Department, Hospital de Sabadell, Sanitary Consortium of Parc Taulí, Universitat Autònoma de Barcelona. Parc Tauli s/n, 08208 Sabadell (Barcelona), Spain; 11 Rheumatology Department, Hospital de Sabadell, Sanitary Consortium of Parc Taulí, Universitat Autònoma de Barcelona. Parc Tauli s/n. 08208 Sabadell (Barcelona), Spain; 12 Primary Healthcare Centre Corbera de Llobregat, Catalan Health Institute. C/ Buenos Aires 9, 08757 Corbera de Llobregat (Barcelona), Spain; 13 Emergency Department, University Hospital of Bellvitge, Catalan Health Institute. University of Barcelona. C/ de la Feixa Llarga s/n, 08907 L'Hospitalet de Llobregat (Barcelona), Spain; 14 Primary Healthcare Research Support Unit Metropolitana Nord, Catalan Health Institute-IDIAP Jordi Gol. Rambla 227, 08223 Sabadell (Barcelona), Spain; 15 Primary Healthcare Centre Creu Alta, Catalan Health Institute. C/ Castellar del Vallès 222, 08280 Sabadell (Barcelona), Spain; 16 Primary Healthcare Centre Bon Pastor. C/ Mollerussa s/n, 08030 Barcelona, Spain; 17 Primary Healthcare Centre Serraparera, Catalan Health Institute. Avda Diagonal s/n, 08290 Cerdanyola del Vallès (Barcelona), Spain; 18 Botnar Research Centre, Nuffield Department of Orthopaedics, Rheumatology and Musculoskeletal Sciences. University of Oxford. Oxford OX3 7LD. UK; 19 Internal Medicine Service, IMIM-Hospital del Mar. Department of Medicine, Universitat Autònoma de Barcelona. Psg Marítim 25, 08003 Barcelona. Spain

## Abstract

**Background:**

Age-related bone loss is asymptomatic, and the morbidity of osteoporosis is secondary to the fractures that occur. Common sites of fracture include the spine, hip, forearm and proximal humerus. Fractures at the hip incur the greatest morbidity and mortality and give rise to the highest direct costs for health services. Their incidence increases exponentially with age.

Independently changes in population demography, the age - and sex- specific incidence of osteoporotic fractures appears to be increasing in developing and developed countries. This could mean more than double the expected burden of osteoporotic fractures in the next 50 years.

**Methods/Design:**

To assess the predictive power of the WHO FRAX™ tool to identify the subjects with the highest absolute risk of fragility fracture at 10 years in a Spanish population, a predictive validation study of the tool will be carried out. For this purpose, the participants recruited by 1999 will be assessed. These were referred to scan-DXA Department from primary healthcare centres, non hospital and hospital consultations. Study population: Patients attended in the national health services integrated into a FRIDEX cohort with at least one Dual-energy X-ray absorptiometry (DXA) measurement and one extensive questionnaire related to fracture risk factors. Measurements: At baseline bone mineral density measurement using DXA, clinical fracture risk factors questionnaire, dietary calcium intake assessment, history of previous fractures, and related drugs. Follow up by telephone interview to know fragility fractures in the 10 years with verification in electronic medical records and also to know the number of falls in the last year. The absolute risk of fracture will be estimated using the FRAX™ tool from the official web site.

**Discussion:**

Since more than 10 years ago numerous publications have recognised the importance of other risk factors for new osteoporotic fractures in addition to low BMD. The extension of a method for calculating the risk (probability) of fractures using the FRAX™ tool is foreseeable in Spain and this would justify a study such as this to allow the necessary adjustments in calibration of the parameters included in the logarithmic formula constituted by FRAX™.

## Background

### Epidemiology of osteoporotic fractures

Osteoporosis is an asymptomatic disease until it is complicated by a bone fracture occurring without trauma or after a minimum trauma. It is the most common bone disease in humans and represents an important health care problem in developed countries. The high incidence of osteoporosis worldwide and its main complication, osteoporotic fractures, also known as fragility fractures, have been recognised for more than 20 years [1]. One of the first meta-analyses on fracture risk published in 1996 demonstrated the association between bone mineral density (BMD) and the risk for osteoporotic fracture [2].

The probability of a woman with menopause presenting an osteoporotic fracture during the remainder of her life (the most frequent are; vertebral, forearm, humerus or hip) surpasses even the risk of having breast cancer, with this probability being approximately 40% higher in developed countries and very close to the risk of coronary disease in the same countries [3].

According to the recent guidelines by the American College of Physicians for the screening of osteoporosis in males, this disease is considered to be underdiagnosed and under-treated, perhaps due to the relatively lower frequency. A 60-year-old white man has a 25% risk of having an osteoporotic fracture during his lifetime, with even more severe consequences than in women [4]. Indeed, the post-hip fracture mortality at one year in men is double that in women [4]. The influence of fragility fractures on the quality of life of both men and women has also been widely reported [5].

According to data estimated in subjects over the age of 50 years in Europe in the year 2000, 620,000 new hip fractures, 575,000 shoulder fractures, 250,000 proximal humerus fractures and 620,000 symptomatic vertebral fractures were reported, representing almost 35% of the fractures described in the world [6]. The direct costs of osteoporotic fracture in Europe are of around a total of 36 billion Euros per year [7].

The greatest clinical relevance of osteoporosis is constituted by osteoporotic fractures, and these are implicated in the increase in morbimortality and loss of quality of life attributable to this disease. Thus, attention must be focused on the identification of patients with a high risk of fragility fracture [8], than on the identification of those with osteoporosis, diagnosed exclusively by densitometry.

Although BMD (measured by densitometry) is an important component of fracture risk, several other risk factors have also been demonstrated to greatly contribute to the risk of fracture and should be taken into account when performing a global evaluation of risk [8].

### Clinical determinants of osteoporotic fracture

In the last years different studies have been carried out with the aim of identifying the clinical risk factors which may be used in the search for therapeutic strategies, with or without the use of densitometry [9].

The last version of the European guidelines for the diagnosis and treatment of osteoporosis in postmenopausal women published in 2008 [10] proposes the strategy of evaluation together the results of densitometry and clinical risk factors of fracture to decide which diagnostic and therapeutic interventions to implement.

### The FRAX ™ tool, a useful tool for clinical practice

In 2008, the WHO published a new promising tool for the evaluation of absolute risk of fragility fracture: the FRAX™ tool [11], WHO fracture risk assessment tool. This is a scale including 11 of the clinical risk factors which have demonstrated a strong association with the incidence of fracture in previous studies according to the WHO experts. Factor number 12 in this scale also includes a single value of Dual-energy X-ray absorptiometry (DXA) central bone densitometry: the T-score of the femoral neck. An introducing these data of a patient provided in the form of the FRAX™ website, an individualised calculation of the percentage of prediction of absolute risk of: (a) major osteoporotic fracture (clinical vertebral, hip, forearm or humeral fracture) and (b) hip fracture in the following 10 years may be made [11].

To develop the logarithmic formula of FRAX™, were included parameters from different European cohorts from the EVOS study focused on vertebral fractures [12]. As representatives of the Spanish population were included people from Oviedo and other three Spanish cities. However, they had very low rates of response: in some cases were less than 8%, with a total number of subjects potentially insufficient to be representative of Spanish population [13].

On the other hand, it should be pointed out that as recommended in the description of the FRAX™ tool, this scale should be developed and validated in each country. Cost-effectiveness studies are also recommended with the data from each country to obtain an approximation of the cost which each country is willing to accept as reasonable for the prevention of fragility fractures.

It is therefore reasonable for the first step before the generalised use of the FRAX™ scale in the medical offices of our country to carry out the validation of this scale in a larger cohort made up of the patients usually attended at the different health care levels in which diagnostic; treatment and follow up interventions for osteoporosis are undertaken. On the other hand, recent evidence [14-17] also recommend the evaluation of other risk factors related to low mass and risk of fragility fracture and not considered in the FRAX™ tool when assessing fracture risk such as the presence of chronic obstructive pulmonary disease (COPD), the use of some drugs such as aromatase inhibitors (increasingly more frequent in women treated for breast cancer), daily calcium intake and usual physical activity which are related to bone mass and risk of fragility fracture.

### Falls and fragility fractures

Fragility fractures are defined as those which occur after non major impact produced by a fall from a height of less than that of the patient with no added inertia to that of the displacement of its foot when walking.

Since more than 20 years ago studies have demonstrated the importance of falls on the incidence of new fractures in predisposed subjects, with a strong association between the number of falls and fracture. This association is even more important in patients over the age of 75 years than the classical association described between osteoporosis and fracture [16]. Different variables related to the greater risk of falls have also been reported such as factors of the individuals themselves (muscular strength of the lower extremities, equilibrium or postural competence, difficulties in vision, cognitive deterioration), purely environmental factors (home lighting, rugs, pets...) and iatrogenic factors (different groups of drugs, drug combinations) [18-21].

Despite these evidence on the potential influence of falls on the occurrence of fragility fractures, they have not been included as risk factors in the FRAX™ tool for the determination of absolute fracture risk at 10 years, probably due to the publication of studies with contradictory long term results which impede consistent establishment of their association.

The importance of being able to determine the association between the number of falls and the appearance of fragility fracture may be established by the possibility of their prevention, thereby reducing the risk of falling. Different studies have presented good results with different training techniques and a recent Cochrane review [22] provides measures of the potential benefit of interventions such as programmes of multidisciplinary detection and intervention (RR 0.73;CI 95%: 0.63-0.85), muscular strengthening and balance retraining (RR 0.80; CI 95%: 0.66-0.98), evaluation and modification of risks at home (RR 0.66; CI 95%; 0.54-0.81), withdrawal of psychotropic drugs (RR 0.34; CI 95%: 0.16-0.74) and a 15-week intervention of Tai Chi group exercises (RR 0.51; CI 95%: 0.36-0.73) among others.

The latest guidelines published in our country recommend intervention related to the risk of fall in subjects with osteoporosis according to a maximum grade of evidence (SEIOMM Guidelines 2008, AATRM Guidelines) [23,24].

The high incidence of falls in the elderly (30% of subjects over the age of 65 years living at home fall every year) [22], as well as the associated morbidity and the tests available demonstrate the relevance of a study such as this to establish their association and determine the need for their inclusion as important and preventable risk factors in tools such as FRAX™ to assess the absolute risk of osteoporotic fractures.

FRAX™ is a tool which is evolving and in the future may become a commonly used tool in medical centres in our country, especially in Primary Care (PC) in which the greatest number of subjects with osteoporosis is attended and where programmes of prevention of osteoporotic factors may be carried out. This is another argument reinforcing the need for urgent validation of this scale in our country, and it is the main objective of this study.

## Objectives

### Main objective

To determine the predictive validity of the WHO FRAX™ risk scale to identify subjects with the greatest absolute risk of fragility fracture in the next 10 years in a Spanish population in a clinical cohort designed to promote the study of different risk factors of presenting osteoporotic fractures.

### Secondary objective

To analyse the association between clinical and environmental risk factors (number of falls, exposure to drugs, dietary calcium intake) and the occurrence of osteoporotic fracture in a susceptible Spanish population.

## Methods/Design

Study of predictive evaluation of a tool to assess the risk of osteoporotic fracture through the follow up of a cohort initiated in 1999.

## Study population and enrolment procedures

This multicentre study is carried out by family practitioners and other specialists who refer patients to the same reference centre for undertaking BMD. The criteria for referral follow the recommendations of the WHO of not performing a population screening but to select cases among those of greatest risk of having osteoporosis and subsequent osteoporotic fractures or the follow up and control of patients already receiving treatment.

*The FRIDEX cohort (Factors of fracture risk and central bone densitometry)*. This cohort is constituted of men and women referred by general practitioners and specialists for undergoing central bone densitometry by *Dual-energy X-ray absorptiometry *(DXA) for the initial study of osteoporosis or treatment follow up, who accept to answer an extensive questionnaire on risk factors (RF) for osteoporotic fracture (family history of osteoporosis and hip fracture, clinical risk factors and lifestyle habits related to diet and toxic substances) [see Table 1]. This cohort was initiated in 1999 at the Bone Densitometry Unit of the Department of Nuclear Medicine of the University Hospital Vall d'Hebrón in Barcelona and at the end of 2009 had included 25,783 persons of both genders who had undergone a total of 41,849 DXA and questionnaires on RFs.

**Table 1:** Total FRIDEX (2000-2010 years) cohort description

		Total	Men	Women
Cases		25,783	2,349	23,434

		N (SD)	N (SD)	N (SD)

Age		61.2 (10.2)	65.0 (10.8)	60.8 (10.1)

Weight		68.1 (12.2)	75.3 (13.3)	67.4 (11.9)

Height		155.6 (7.4)	165.7 (7.2)	154.6 (7.4)

		N (%)	N (%)	N (%)

Parental Osteoporosis or Fracture	Yes	4,220 (16.4%)	153 (6.5%)	4,067 (17.4%)
	No	21,524 (83.6%)	2,192 (93.5%)	19,332 (82.6%)

Parental Hip Fracture	Yes	166 (6.3%)	10 (2.6%)	156 (6.9%)
	No	2,462 (93.7%)	372 (97.4%)	2,090 (93.1%)

Previous Fractures	Yes	6,865 (26.6%)	837 (35.6%)	6,028 (25.7%)
	No	18,918 (73.4%)	1,512 (64.4%)	17,406 (74.3%)

Current Prescriptions	Yes	13,928 (54.0%)	1,048 (44.6%)	12,880 (55.0%)
	No	11,855 (46.0%)	1,301 (55.4%)	10,554 (45.0%)

Since the beginning of the study verbal informed consent to participate in the cohort was obtained from all the patients and an extensive questionnaire on clinical risk factors was carried out. The data collected is stored in a specific database (DB) for this cohort.

Informed consent to participate is requested in the reference centre and a questionnaire on risk factors (QRF) for osteoporotic fractures is given during the visit and anthropometric parameters are determined. Ten years after the first QRF and DXA the patients are asked to answer a phone survey (See additional file 1) to know the evolution of the study variables and outcomes (fragility fractures).

## Study population

Urban setting. Primary care (PC), extrahospitalary (E) and hospital specialties (H).

Integrants of the FRIDEX cohort. Randomised sample (simple computerised randomisation stratified by sex) of men and women from 40 to 90 years of age in the FRIDEX cohort for 10 years since the baseline DXA and QRF. At the end of 2009 this sub-cohort included 5,813 persons recruited from January 1 to December 31, 2000.

## Eligibility Criteria

A total of 3,684 subjects were randomised, 9.3% being males to maintain the original proportion of the global study cohort.

## Inclusion criteria

The study subjects were Caucasians, ≥ 40 and ≤ 90 years of age at the time of inclusion in the FRIDEX cohort, understood and spoke the Spanish language, were able to respond to the initial and/or follow up telephone questionnaire (TQ) and accepted to participate in the study providing the corresponding informed consent. Physically or psychically handicapped patients were included if the relatives or care providers accepted to answer the TQ.

## Exclusion criteria

Subjects < 40 or > 90 years of age at the time of the first DXA and QRF were excluded since FRAX™ does not allow the calculation of the adjusted risk outside this age range. Patients with physical or psychic limitations impeding their participation and whose relatives did not accept to respond to the TQ were excluded as were those with Paget's disease, cancer with bone involvement or disease which may simulate osteoporosis (i.e. myeloma). Patients of ethnic groups other than Caucasian were not included since other studies have demonstrated different risk characteristics. Patients not providing consent to respond to the TQ and those without a telephone to contact or did not respond after 3 calls made at different times according to the procedure manual were also excluded from the study.

**Figure 1:**
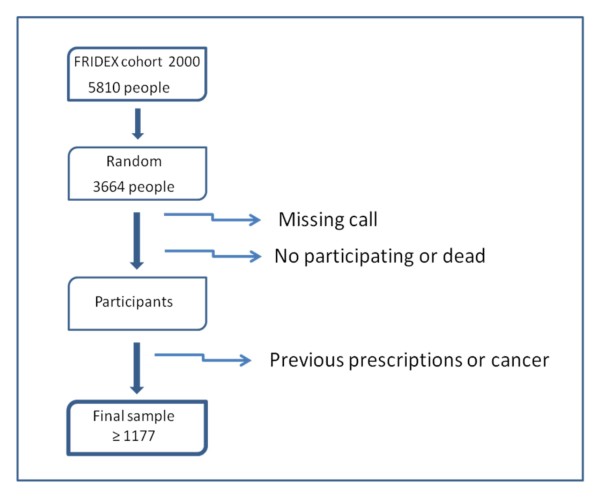
**FLOW CHART study**. Participant's selection.

Sample Size (figure 1):

For the main objective (predictive validation of FRAX™ it has been calculated that a sample of 1,070 individuals are needed in a bilateral contrast to guarantee that the sample estimates the percentage of incidence of new fractures with a precision of 3%. If an annual loss rate of 1% during the 10 years of follow up of the study is considered, a sample of 1,177 subjects is required. In the pilot study carried out in April 2009 in a randomised sample of 149 cases with three telephone calls / person, 47 persons could not be contacted (31.5%). One hundred two (68.5%) were contacted, of which 3 (4.9%) living patients and the relatives of 2 dead patients refused to participate. Cases receiving anti-osteoporotic drugs and/or those with cancer (35 people) and 6 males were excluded. Information was obtained on data at 10 years in 97 of the 149 cases. A total of 3,664 individuals should therefore be contacted, thus our population of 5,813 subjects potentially eligible guarantees the necessary sample size.

Finally, in the pilot study of 149 patients and after fulfilling the exclusion criteria we obtained a sample of 56 (37.6%) of the 149 cases. With more conservative calculations, a maximum of 3,056 subjects should be contacted plus 20% for safety. Thus 3,664 persons should be contacted, therefore our originally recruited population of 5,813 subjects greater than 40 years of age included in the cohort until the end of 2000 sufficiently guarantees the necessary sample size. Consequently, 3,664 subjects were randomly selected from the 5,813 patients included in the cohort during the year 2000.

A sample will be obtained by months in the year 2000. Ordering will be performed using randomised numbers for each month and the calls will be made in this order until the required number is achieved. The approximate calculation is of 317 cases / month with a range from 190 to 395 cases in the different months of 2000, and the calls will be made until the figure calculated per month is met.

## Overview of outcome measurements

### Data collection

The baseline variables of both BMD and QRF were collected at the time of inclusion (2000). The follow up variables (fractures and incidental falls) will be collected by telephone questionnaire (TQ) during 2010 and the beginning of 2011 to complete the 10 years of follow up. The TQ will collect data regarding the fractures occurring from the time of inclusion until the date of the TQ as well as other information on known factors of fracture risk.

### Baseline variables

These include variables related to the patient: Demographic (date of birth, sex), anthropometric (weight, height, body mass index).

BMD measurement will be determined by central DXA according to the 2004 and 2007 recommendations of the International Society for Clinical Densitometry ISCD (available at: http://www.iscd.org/Visitors/positions/OfficialPositionsText.cfm) for the interpretation of the results using a Lunar GE model "Prodigy Advance" densitometer with 11.4 software and with BMD and T-score determination with NHANES III references. The densitometry diagnostic criteria used are the 1994 WHO criteria which classify the results into 3 groups according to the levels of BMD values of the femoral neck: normal (T-score > -1), osteopenia (T-score between -2.5 and -1) and osteoporosis (T-score ≤ -2.5).

Additional file 1 shows the clinical factors of fracture risk analysed with the structured questionnaire (QRF), dietary intake of calcium and drugs use.

The estimated absolute risk of fracture at 10 years according to the FRAX™ tool is determined through the official web site (available at: http://www.shef.ac.uk/FRAX). The calculations of probability of fracture with or without the T-score will be analysed in parallel by two blinded investigators (patients anonymised and assigned an alphanumeric code). On the appearance of any difference a third and fourth blinded investigator will analyse the results and will recalculate the case.

### Follow up variables

These variables include the appearance of incidental fracture in the last 10 years (dependent variable): the telephone questionnaire will be carried out with a telephone call made after 10 years of follow up. All the fractures will be confirmed through medical records and/or consultations to the health care centres after receiving authorisation from the participants. In all cases the follow up at 10 years will be completed in these subjects. In cases of death, the data related to the cause of death and the appearance of fractures will be requested from the relatives and by record checking.

The number of falls during the last year will be determined with the TQ. Review of the literature has shown different ways to analyse the falls occurring in the study subjects. The most frequently used method is considered in this study which asks about the number of falls during the year prior to the interview and whether a fracture was produced in any of the falls. Other variables to be collected during this period are: the appearance of important diseases, the taking of osteopenic drugs and the use of walking aids. Additional file 2 shows the telephone questionnaire.

### Analysis plan

The characteristics of the population will be described according to univariate descriptive analysis. Simple comparisons of the baseline characteristics will be made among the participants and non participants of the cohort. The Chi-square test will be used to evaluate the association between qualitative variables. The Student's t-test or, if necessary, its non parametric equivalent, the Mann-Whitney U test, will be implemented to evaluate the differences in the distribution of a quantitative variable according to the categories defined by a binary exposure. To assess the differences in the distribution of a quantitative variable according to the categories defined by a categorical variable with more than 2 categories, ANOVA analysis of variance or its corresponding non parametric test (Kruskal-Wallis) will be used.

For the predictive validation of the FRAX™ tool, the appearance of the first fracture occurring during the follow up period will be taken into account. The validation of the results obtained with the FRAX™ will be performed with the Hosmer-Lemeshow test and the calculation of the ROC curve. This test divides the participants into groups (normally 10) based on their estimated risk of fracture (FRAX™) and confirms that each group presents a number of cases of incidental fractures adjusted to the predicted number. On the other hand, the ROC curve considers the scale of FRAX™ risk as a diagnostic test of the presence of future fractures and as such leads to different calculations of sensitivity and specificity changing the cut off point selected. Finally, both probabilities (sensitivity and the complementary of specificity, or 1- specificity) will be graphically represented the curve. The shape of the curve is a visual indicator of the quality of the diagnostic test.

To know the distribution of the factors associated with fracture according to age and sex, bivariate combinations will be used with the Chi-square test among categorical variables and the Student's test among quantitative and categorical variables. To model the number of fractures occurring in our dataset which is, in fact, a count over time, Poisson regression will be used, which is what is precisely required for this type of variables and was used for the creation of the FRAX™ scale. All the statistical tests will be undertaken with a confidence interval of 95% and with the use of the 17th or latest version available of the SPSS statistical package.

### Study limitations, potential limitations and biases

Since the FRIDEX cohort is constituted by subjects requiring a DXA scan (according to their physician), it likely that the recruited population will be at a baseline risk greater than that of the general population. Nonetheless, descriptive analysis of the population of this cohort indicates that the percentage of 32.3% of persons with densitometry osteoporosis is very similar to that reported in the literature for women of 50 years of age. Our results may therefore be extrapolated to a population in which the physician is evaluating the risk of low bone mass or fracture (case finding) which is, furthermore, the population recommended for investigation by the WHO.

The QRF used includes the variables of the FRAX™ scale and is complemented by the follow up telephone questionnaire on fractures, falls and new medications prescribed as well as diseases developed in the last 10 years.

The variable "number of falls" was not collected at the beginning of the FRIDEX cohort. Therefore, it may only be considered as an outcome variable in the subgroup of cases with incidental fractures posterior to the collection of this variable. Nonetheless, according to the opinion of the external assessor and the research team, this variable is highly related to the risk of fracture and thus, should be collected and taken into account in this study.

To minimise the effect of possible losses which may imply bias (given the morbimortality associated with fractures and the possible dropouts over 10 years), notable increases in the sample size have been considered necessary such as the cases to be localised among those with a contact telephone number. We believe that this will minimise the losses to follow up or refusal to participate for several reasons: information will be collected by telephone to avoid the difficulties of post questionnaires, which have low response rates in our setting; in addition the participants in the FRIDEX cohort had already accepted to participate in the QRF and almost all of the persons contacted in the pilot study accepted to answer follow-up survey.

There may be a bias in the collection of the information on incidental fractures which is collected based on the patient self-report. Nevertheless, in this study all the new fractures detected will be contrasted with the corresponding medical reports or by consultation with the physicians. Thus silent vertebral fractures will be scarcely detected but the symptomatic vertebral fractures, which are those included in the prediction of the FRAX™, will be collected. In addition, this is the usual method used in large epidemiological studies. The possible limitations inherent to data collection by telephone will be minimised with interviewer training among personnel with health care background through the improvement detected in the pilot study and by the incorporation of potential improvements thereafter.

The study has been approved by the ethical committee of the Clinical Research Ethics Committee of the Vall d'Hebron University Hospital (Barcelona, Spain). Additional file 3 shows the timing of the project.

## Discussion

Since more than 10 years ago numerous reports have recognised the importance of other risk factors for new osteoporotic fractures in addition to low BMD. This new evidence has modified the conception of the utility of BMD as the gold standard or as an added element for decision making in the management of osteoporosis in primary health care settings.

The FRAX™ tool was published by the WHO in 2008 and was created to establish a calculation of the probability of absolute risk of osteoporotic fracture at 10 years. This was initially based on the analysis of thousands of person included in several cohorts in Europe [12,13]. Since its publication this tool has also been used to know the risk of fracture in other population cohorts in Europe, the United States of America, Canada, and Japan [25-30]. Likewise, since its publication this tool has undergone new adjustments and calibrations for different populations [30]. On the other hand, some studies have also recently been published, in which the cases of fracture estimated or expected by the FRAX™ tool were significantly lower than the cases of incidental fractures actually observed in the 10 years of follow up [31].

In the case of the Spanish population included in the FRAX™ tool, there are some doubts as to their representativeness because of the scarce response of original study and scarce global number of patients included [12,13], which could represent problems of external validity and should be contrasted with new studies of large cohorts over long periods of follow up to allow the establishment of epidemiological relationships adjusted to each population.

The extension of a method for calculating the risk (probability) of fractures using the FRAX™ tool is foreseeable in Spain similar to what is occurring in other countries and this would justify a study such as this to allow the necessary adjustments in calibration of the parameters included in the logarithmic formula constituted by FRAX™.

From the point of view of validation and economic analysis of the FRAX™ tool, the National Osteoporosis Guideline Group (NOGG) has recently published a calculation with FRAX™ which takes cost-effectiveness to avoid a new fracture in a population in the United Kingdom into account [15]. This is one of the first countries to publish studies on the economic cost which means the willingness to pay and cost-effectiveness to avoid a new fracture in its population. These calculations have been based on the calculation of fracture risk using the FRAX™ tool and determined clinical risk factors.

It can be expected that other counties will establish the same parameters with economic evaluation derived from the application of the FRAX™ tool. A study of the diagnostic validation of the FRAX™ tool is necessary as the first step to establish the criteria of cost-effectiveness and the number of cases to treat to avoid the appearance of new osteoporotic fractures.

Studies in large cohorts over long periods of follow up have allowed epidemiological associations to be established, and although numerous studies have demonstrated the independent association of clinical and environmental risk factors with low BMD, some associations with fractures in populations in different geographical zones are pending thus, the need for extensive epidemiological studies in the Spanish population.

One of the few studies published with the FRAX™ tool in Spain did not provide new knowledge on the idealness of the cohorts represented [32], thereby justifying a study such as the present which will allow the necessary calibrations and adjustments of the parameters included in the logarithmic formula constituted by the FRAX™. At the same time, the scientific community requires a relatively easy and agile system to determine when a DXA should be requested and/or when treatment for osteoporosis should be initiated based on reliable predictive models similar to those already implemented in the daily routine for the prevention of cardiovascular events.

The WHO has recommended that prospective studies should be performed with this methodology in our population. This is therefore a great opportunity to validate and contribute to the determination of its true utility among the collective of physicians, especially in Primary Care, and in the population by focusing on interventions in the cases of greatest risk of fracture.

## Ethics

The study has been approved by the ethical committee of the Clinical Research Ethics Committee of the Vall d'Hebron University Hospital (Barcelona, Spain).

Telephone calls will be made to all the cases of the randomised sample of the cohort. According to the protocol accepted by the reference Clinical Research Ethics Committee [PR registration number (AG) 68/2009], verbal consent will be requested at the onset of the study to continue with the telephone interview, the posterior follow up calls and to compare the fractures in the medical records.

The investigators guarantee and will be responsible for data confidentiality. The clinical data will be introduced into a computerised database in which the patients are identified by an anonymised code. A parallel database will be created containing the data of patient affiliation and the corresponding relational code. Only the Principal Investigator and the study data manage will have access to this database.

## List of abbreviations

AATRM: Agència Avaluació Tecnologia i Recerca Mèdiques (Catalan Agency for Health Technology Assessment and Research); BMD: Bone Mineral Density; COPD: Chronic Obstructive Pulmonary Disease; DB: Database; DXA: Dual-energy X-ray Absorptiometry; E: Extrahospitalary specialties; FRAX™: Fracture Risk Assessment tool; FRIDEX: Factors of Fracture Risk and Bone Densitometry DXA cohort; H: Hospital specialities; ISCD: International Society for Clinical Densitometry; NAHNES: National Health and Nutrition Examination Survey; NOGG: National Osteoporosis Guideline Group; PC: Primary Care; QRF: Questionnaire of Risk Factors; RF: Risk Factor; RR: Relative Risk; SEIOMM: Sociedad Española Investigacion Osea y Metabolismo Mineral (Spanish Society for Bone and Mineral Research); TQ: Telephone Questionnaire: follow up; WHO: World Heath Organisation.

## Competing interests

The authors declare that they have no competing interests.

## Authors' contributions

RA is the principal investigator, project design and direction, preparation and review of the manuscript. GR coordination field work, preparation and review of the manuscript. GE coordination and management of the cohort, review of the manuscript. AA coordination and analysis of the FRAX™ values, review of the manuscript. MI, NP, MZ, SG, PS, SS, VS, FLE, SO and YF field work, calculation of the FRAX™ values and review of the manuscript. DP statistical analysis and management of the database, review of the manuscript. EG, EC, PT and ADP scientific support and methodological expert, review of the manuscript. All authors read and approved the final manuscript.

## Pre-publication history

The pre-publication history for this paper can be accessed here:

http://www.biomedcentral.com/1471-2474/12/30/prepub

## Supplementary Material

Additional files 1 **Questionnaire on risk factors (QRF)**. Shows the clinical factors of fracture risk analysed with the structured questionnaire (QRF), dietary intake of calcium and drugs use. Click here for file

Additional file 2 **Telephone Questionnaire (TQ)**. Shows the follow-up survey of factors that can influence the number and type of fractures, falls, drugs, and others. Click here for file

Additional file 3 **Timing of the Project**. Shows the work plan, broken down by task and timetable Click here for file
