# Supplementary material for: Prediction of absolute risk of fragility fracture at 10 years in a Spanish population: validation of the WHO FRAX ™ tool in Spain
Source: BMC Musculoskelet Disord. 2011 Jan 28;12:30. doi: 10.1186/1471-2474-12-30 (PMC3224379; doi:10.1186/1471-2474-12-30)
Supplement: Additional files 1 — Questionnaire on risk factors (QRF). Shows the clinical factors of fracture risk analysed with the structured questionnaire (QRF), dietary intake of calcium and drugs use. [file 1471-2474-12-30-S1.DOC]

**Additional File 1. Questionnaire on risk factors (QRF)**

Surname: Name:

Date of birth (dd/mm/jy): / /19

Height (cm):

Weight (Kg):

**A) Family history**

Family history of osteoporosis

No (0) Yes (1) fracture of femur/spine (1)

**B) History of disease**

00.- None

01.- Picture of malabsorption

02.- Imperfect osteogenesis

03.- Rachitis (intolerance to Vit D)

04.- Intolerance to milk products

05.- Long periods of immobilisation

06.- Chronic juvenile arthritis

07.- Anorexia nerviosa

08.- Growth retardation

09.- Diabetes

10.- Hypothyroidism

11.- Hyperthyroidism

12.- Hypogonadism

13.- Cirrhosis

14.- Chronic renal insufficiency

15.- Hyperparathyroidism

16.- Endometriosis

17.- Hyperprolactinaemia

18.- Gynaecological neoplasm (breast, ovarian, uterine)

19.- Gastrectomy or extensive intestinal resection

20.- Chronic bronchial asthma

21.- Rheumatic arthritis

22.- Hypercalciuria

23.- Turner syndrome

24.- Prolonged amenorrhea (induced by exercise)

25.- Prolactinoma

26.- Others

**C) Fractures**

00.- None

01.- Spine

02.- Femur

03.- Humerus

04.- Pelvis

05.- Forearm/wrist

06.- Ribs

07.- Fingers/toes

08.- Others

**D) Physical activities/ Physical and toxic habits**

00.- Sedentary activity

01.- Activity derived from work

02.- Deambulation (walks, golf, jogging )

03.- Gymnasium

04.- Swimming

05.- Tennis

06.- Others

**Intensity of physical activity**

0.- Occasional or 1h/week

1.- < 5h/week

2.- > 5h/week

**Smoking**

0.- Non smoker

1.- Ex smoker

2.- Smoker nº cigarettes/day

**Alcohol**

0.- Occasionally

1.- Usually

**Beer intake :**

200 cc bottle = 8 gr. Alcohol

300 cc bottle = 12 gr. Alcohol

**Wine, sparkling wine intake:**

1 glass of wine with water (50 cc) = 5gr

1 glass of wine without water (100 cc) = 10gr

**Coffee with brandy intake :**

1 glass (20 cc) = 7 gr.

**Spirits:**

1 glass (60 cc) = 9 gr.

**Consumption of liquors:**

1 glass (40 cc) = 14 gr. **Grammes alcohol / day . . . . . . . . .**

**Medications**

**00.- None**

**01.- Oral contraceptives**

Diane 35, Gynefix, Gynovin, Harmonet, Meliane, Microdial, Microgynon, Minulet, Multiload, Ovoplex, Neogynona, Suavuret, Tri-munulet,T, Triciclor, Trigynovin

**02.- Sterility treatment**

Danatri, Decapeptyl, Gonal-f75, Hcg Lepori, Hmglepori, Neo-Fertinorm, Orgametril, Parlodel, Profasi Hp, Progevera, Puregon, Synarel, Zoladex

**03.- Treatment of Endometriosis**

Danatrol, Decapeptyl, Ginecrin Depot, Gynovin, Meliane, Motronidazol, Neogynoma, Orgametril, Ovoplex, Primolut, Progevera, Synarel, Prolongatum, Tricolam, Zoladex

**04.- Corticoides**

Dacortin, Dezacor, Zamene, Urbason

**05.- Thyroid hormones**

Dexnon, Levothyroxine, Tiroxina, Auxina, Cebion, Lopresor, Seloken, Tirodril, Eutirox

**06. - Antacids**

Almax, Alubifar, Bemolan, Dolcopin, Gelodrox, Gelodual, Gelotrisin, Maalox, Magion, Minoton, Secrepat, Unimaalox, Geloalumin, Alcalinos Gelos, Gelotricar

**07.- Anticonvulsants**

**08.- Antidepressants (lithium salts)**

Plenur, Serosat.

**09.- Heparin and anticoagulants**

Sinthrome, Heparin and other Anticoagulants

**10.- Diuretics (thiacidics and others)**

Seguril, Furosemide, Hydrosaluretil, Ameride

**11.- Chemotherapy**

Of more than 5 years (note specific treatments)

**12.- Oestrogens**

Absorlent, Aerodiol, Alcis, Carentil, Cliogan, Conestron, Dermestril, Endomina, Estraderm, Estrdiol, Evopad, Menorest, Meriestra, Novafem, Oestraclim, Estrodose, Ovestinon, Premarin, Progynova, Progynon, Angeliq

**13.- Calcium**

Calcium Sandoz, Caosina, Cimascal, Densical, Calcium chloride, Fortical, Gluconate, Ibercal, Mastical, Natecal, Ossopan, Osteopor, Ostram, Tepoxcal

**14.- Vitamin D**

Hidroferol, Calcivex, Etalpha

**15.- Calcitonin (with or without calcium)**

Calogen, Calsynar, Kalsimir, Miacalcic, Osototal, Osteobion, Sical, Tococaltin, Ucecal, Elcantonina, Ospor

**16.- Fluor**

Multibionta mineral.

**17.- Diphosphonates (with or without calcium)**

Difosfen

**18.- Progesterone**

Progeffik, Progestogel, Utrogestan, Progevera, Colpro

**19.- Oestrogens+ Progestagens**

Activille, Premelle, Absorlent, Trisequens, Progyluton, Eslis, Sequi, Nuvelle, Perifem

**20.- Fluor+ vit D+ calcium**

**21.- Anabolizers**

Deca Durabolin

**22.- Collagen derivatives**

Xicil

**23.- Raloxiphen**

Evista, Optruma

**24.- Tamoxifen**

Tamoxifen, Nolvadex

**25.- Calcium + vit. D**

Calcium Forte D, Cimascal D Forte, Creacal, Disnal, Ideos, Mencalisvit, Natecal D, Osteomerck, Ostine, Veriscal D, Osvical.

**26.- Tibolone**

Boltin

**27.- Alendronate**

Fosamax, Fosavance

**28.- Resindronate**

Actonel, Acrel.

**29.- Pamidronate e. v.**

Aredia, Ibandronato (ev)

**31.- Estrone**

Protelos, Osseor

**32.- Aromatase inhibitors |**

Arimidex (1) Anastrozol / Femara (2) Letrozol / Aromasil (3) Exemestine

1. Non steroidal anastrozoles,
2. Non steroidal letrozol,
3. Steroidal exemestine,

**33.- Other bisphosphonates**

Osteum, Didronel (etidronate), Bonviva (oral), Neridronate,

Bonefos, Mebonate, Nemocalcin (clodronate), Hemocalcin, Didronel

**34.- Paratohormone analogues**

Teriparatoideas Pth, Preotac, Forsteo

**35.- Zoledronic**

Zometa (ev), Aclasta (ev)

**E) Gynaecological history**

Age at menarche

Number of gestation greater than 6 months

Total duration of lactation in months

Complete anexectomy or total hysterectomy: No (0), Yes (1)

Age at menopause (date of last period)

**F) Questionnaire on nutritional calcium: (taken from INDICAD)**
