# Supplementary material for: Prediction of absolute risk of fragility fracture at 10 years in a Spanish population: validation of the WHO FRAX ™ tool in Spain
Source: BMC Musculoskelet Disord. 2011 Jan 28;12:30. doi: 10.1186/1471-2474-12-30 (PMC3224379; doi:10.1186/1471-2474-12-30)
Supplement: Additional file 2 — Telephone Questionnaire (TQ). Shows the follow-up survey of factors that can influence the number and type of fractures, falls, drugs, and others. [file 1471-2474-12-30-S2.DOC]

**Additional File 2. Telephone Questionnaire (TQ)**

|  | **Telepone questionnaire** | | | **FRAX-FRiDEX study** | |  |  | **Responses** | **Var Categories** | | | | |  |  |  | |
| --- | --- | --- | --- | --- | --- | --- | --- | --- | --- | --- | --- | --- | --- | --- | --- | --- | --- |
|  | Code |  | Mr/Mrs. |  |  | | | |  | | | | |  |  |  | |
|  | Call 1 |  | Call 2 |  | Call 3 |  |  |  | 1^st^ call: 1/2^nd^: 2/3^rd^: 3 | | | | | |  |  | |
|  | Date of birth | | / / | | Date of questionnaire | | / / |  | Date | | | | |  |  |  | |
|  |  |  |  |  |  |  |  |  |  | | | | |  |  |  | |
|  | **A) CASES OF HIP FRACTURE IN THE PARENTS of the patients** | | | | |  |  |  |  | | | | |  |  |  | |
| 1 | Did your **mother or father** have a hip fracture after the age of 50 years? | | | | | | |  | (Yes 1/N 0) | | | | |  |  |  | |
| 2 | If the answer is YES, please indicate MOTHER or FATHER. | | | | | | |  | MOTHER 1/FATHER 2 | | | | | |  |  | |
| 3 | Approximately what age was SHE/HE? | | | | | | |  | Age in years | | | | |  |  |  | |
| 4 | Approximately what year did this happen? | | | | | | |  | Year with 4 numbers | | | | | |  |  | |
|  |  |  |  |  |  |  |  |  |  | | | | |  |  |  | |
|  | **B) Personal OLD FRACTURES since the previous questionnaire.** | | | | |  |  |  |  | | | | |  |  |  | |
| 5 | Have you (patient) had a fracture since the DXA TEST? **(If NOT go to section C)** | | | | | | |  | Yes 1/No 2 | | | | |  |  |  | |
| 6 | What bone did you break? | | | | | | |  | Name | | | | |  |  |  | |
| 7 | What date did the fracture occur? | | | | | | |  | Date | | | | |  |  |  | |
| 8 | Was it caused by an important accident such as a traffic accident or something similar? | | | | | | |  | YES 1/No 2 | | | | |  |  |  | |
| 9 | If it was due to a fall was it while walking (include: curb-step)/ from bed?/higher height such as stair, etc? | | | | | | |  | Standing or walking 1/  bed or chair 2/  higher 3 | | | | | | | |  |
| 10 | In what hospital or primary care centre were you attended? | | | | | | |  | Name | | | | |  |  |  | |
| 11 | Was the fracture confirmed by x-ray? | | | | | | |  | YES 1/No 2 | | | | |  |  |  | |
| 12 | Do you have the x-ray or report at home? | | | | | | |  | YES/No 2 | | | | |  |  |  | |
|  |  |  |  |  |  |  |  |  |  | | | | |  |  |  | |
|  | **C) BONE FRACTURES IN THE LAST 12 MONTHS.…..** | | | |  |  |  |  |  | | | | |  |  |  | |
| 13 | Have you had any **fracture** (in the last year)? **(If NOT go to section D)** | | | | | | |  | Yes 1/No 2 | | | | |  |  |  | |
| 14 | What bone did you break? | | | | | | |  | Name | | | | |  |  |  | |
| 15 | What date did the fracture occur? | | | | | | |  | Date | | | | |  |  |  | |
| 16 | Was it caused by an important accident such as a traffic accident or something similar? | | | | | | |  | Yes 1/No 2 | | | | |  |  |  | |
| 17 | If it was due to a fall was it while walking (include: curb-step)/ from bed?/higher height such as stair, etc? | | | | | | |  | Standing or walking 1/  bed or chair 2/  higher 3/ | | | | | | | |  |
| 18 | In what hospital or primary care centre were you attended? | | | | | | |  | Name | | | | |  |  |  | |
| 19 | Was the fracture confirmed by x-ray? | | | | | | |  | Yes 1/No 2 | | | | |  |  |  | |
| 20 | Do you have the x-ray or report at home? | | | | | | |  | Yes 1/No 2 | | | | |  |  |  | |
|  |  |  |  |  |  |  |  |  |  | | | | |  |  |  | |
|  | **D) FALLS in the last 12 months** | |  |  |  |  |  |  |  | | | | |  |  |  | |
| 21 | Have you had any **fall** in the 12 months? **(If NOT go to section E)** | | | | | | |  | Yes 1/No 2 | | | | |  |  |  | |
| 22 | Do you have any disease or limitation which might favour falls? | | | | | | |  | Yes 1/No 2 | | | | |  |  |  | |
| 23 | If the answer is YES, please specify? | | | | | | |  | Name | | | | |  |  |  | |
| 24 | Were you walking (include: curb-step)/did you fall from bed? from a higher height such as the stairs, etc? | | | | | | |  | Standing or walking 1/  bed or chair 2/  higher 3/ | | | | | | | |  |
| 25 | Where did this happen? At home/the street/shop/in the country or another zone where accidents may occur? | | | | | | |  | home 1/ street 2/ shop 3/  country or other hilly area 4/ | | | | | | | |  |
| 26 | If at home, in what part of the house did this occur? | | | | | | |  | Name | | | | |  |  |  | |
| 27 | How many times have you fallen in the last 12 months? (For any reason) | | | | | | |  | nº | | | | |  |  |  | |
| 28 | How many of these falls have been attended in health care centres with x-ray? | | | | | | |  | nº | | | | |  |  |  | |
|  |  |  |  |  |  |  |  |  |  | | | | |  |  |  | |
|  | **E) RADIOGRAPHIES since the previous questionnaire** | | |  |  |  |  |  |  | | | | |  |  |  | |
| 29 | Have you ever had an x-ray of the chest or back? | | | | | | |  | Yes 1/No 2 | | | | |  |  |  | |
| 30 | If the answer is YES, do you have it at home, at the Primary Care Centre/hospital? | | | | | | |  | home 1/PCC 2/Hospital 3 | | | | | |  |  | |
|  |  |  |  |  |  |  |  |  |  | | | | |  |  |  | |
|  | **OSTEOPOROSIS** | |  |  |  |  |  |  |  | | | | |  |  |  | |
|  | **F) TAKIG MEDICATION FOR OSTEOPOROSIS** | | | |  |  |  | Drug 1 | Drug 2 | | | | |  |  |  | |
| 31 | Do you currently take any medication to treat or prevent osteoporosis? | | | | | | |  |  | | | | | SI 1/No 2 |  |  | |
| 32 | If the answer is YES, since when have you been taking it in months? | | | | | | |  |  | | | | | nº meses |  |  | |
| 33 | If the answer is YES, can you tell me the brand name? | | | | | | |  |  | | | | | nombre |  |  | |
| 34 | If the answer is NO, have you ever taken anything? | | | | | | |  |  | YES 1/No 2 |  |  |  | | | | |
| 35 | Can you tell me the brand name of the last? | | | | | | |  |  | | | | | nombre |  |  | |
| 36 | If you had to stop taking it was it due to intolerance?/because your doctor told you to/ or for your own rehaznos? | | | | | | |  |  | | | | | Intolerancia 1/ por su Dr 2/Cuenta propia 3 | | |  |
| 37 | How long did you take it in months? | | | | | | |  |  | | | | | nº meses |  |  | |
| 38 | When did you last take it? | | | | | | |  |  | | | | | fecha |  |  | |
|  |  |  |  |  |  |  |  |  |  | | | | |  |  |  | |
|  | **G) HAS NEW DISEASES since previous questionnaire (seek rheumatic diseases, cancer)** | | | | | |  |  |  | | | | |  |  |  | |
| 39 | Have you had any important disease or operation? | | | | | | |  | Yes 1/No 2 | | | | |  |  |  | |
| 40 | If the answer is YES, what? |  |  |  |  |  |  |  | name | | | | |  |  |  | |
|  |  |  |  |  |  |  |  |  |  | | | | |  |  |  | |
|  | **H) TAKING ANOTHER NEW MEDICATION (continued since previous questionnaire (CORTICOIDES, PPI, SSRI)** | | | | | | |  |  | | | | |  |  |  | |
| 41 | Have you started to take any new chronic medication in the last 12 months (Y/N) | | | | | | |  | Yes 1/No 2 | | | | |  |  |  | |
| 42 | If the answer is YES, give brand name - 1º? | | | | | | |  | name | | | | |  |  |  | |
| 43 | If the answer is YES, give brand name - 2º? | | | | | | |  | name | | | | |  |  |  | |
|  |  |  |  |  |  |  |  |  |  | | | | |  |  |  | |
|  | **I) NEW REVIEWS in the future** | | |  |  |  |  |  |  | | | | |  |  |  | |
| 44 | Do you give us permission to consult your medical history in the PCC/hospital | | | | | | |  | Yes 1/No 2 | | | | |  |  |  | |
| 45 | Do you give us permission to call you and continue participating next year? | | | | | | |  | Yes 1/No 2 | | | | |  |  |  | |
